# Synthesis of Three Dimensional Nickel Cobalt Oxide Nanoneedles on Nickel Foam, Their Characterization and Glucose Sensing Application

**DOI:** 10.3390/s140305415

**Published:** 2014-03-18

**Authors:** Mushtaque Hussain, Zafar Hussain Ibupoto, Mazhar Ali Abbasi, Xianjie Liu, Omer Nur, Magnus Willander

**Affiliations:** 1 Department of Science and Technology, Campus Norrköping, 60174, Linköping University, SE-60174 Norrköping, Sweden; E-Mails: zafar.hussain.ibupoto@liu.se (Z.H.I.); mazhar.ali.abbasi@liu.se (M.A.A.); omer.nour@liu.se (O.N.); magnus.willander@liu.se (M.W.); 2 Department of Physics, Chemistry and Biology, Linköping University, Linköping 58183, Sweden; E-Mail: xianjie.liu@ifm.liu.se

**Keywords:** nickel cobalt oxide nanostructures, nickel foam, glucose sensor, potentiometric method

## Abstract

In the present work, NiCo_2_O_4_ nanostructures are fabricated in three dimensions (3D) on nickel foam by the hydrothermal method. The nanomaterial was characterized by scanning electron microscopy (SEM), X-ray diffraction (XRD) and X-ray photoelectron spectroscopy (XPS). The nanostructures exhibit nanoneedle-like morphology grown in 3D with good crystalline quality. The nanomaterial is composed of nickel, cobalt and oxygen atoms. By using the favorable porosity of the nanomaterial and the substrate itself, a sensitive glucose sensor is proposed by immobilizing glucose oxidase. The presented glucose sensor has shown linear response over a wide range of glucose concentrations from 0.005 mM to 15 mM with a sensitivity of 91.34 mV/decade and a fast response time of less than 10 s. The NiCo_2_O_4_ nanostructures-based glucose sensor has shown excellent reproducibility, repeatability and stability. The sensor showed negligible response to the normal concentrations of common interferents with glucose sensing, including uric acid, dopamine and ascorbic acid. All these favorable advantages of the fabricated glucose sensor suggest that it may have high potential for the determination of glucose in biological samples, food and other related areas.

## 1. Introduction

The synthesis of new nanostructures of transition metal oxides with attractive shape, dimension, size and morphology are of high interest due to their potential usage in fields like material science, physics and chemistry [1–3]. Among the various metal oxides, nickel- and cobalt-based binary oxide materials are of special interest due to their low cost, low toxicity, their high natural abundance and particular morphologies and structures. They also show superior electrochemical performance as a binary metal oxide [4] having a high degree of redox chemistry [5] and electronic conductivity compared to the single phase of nickel and cobalt oxides, therefore NiCo_2_O_4_ can be used as a backbone to support active electrode materials [6]. NiCo_2_O_4_ exhibits a spinel structure in which Ni occupies the octahedral position and Co is spread over both octahedral and tetrahedral positions [7,8]. The synthesis of NiCo_2_O_4_ with a rationally designed nanostructure is imperative if it is to be used as an anode material in high-performance sensor devices. Generally, electrode materials with hierarchical porous structures exhibit many advantageous properties that improve the electrochemical performance of sensors, such as their ability to alleviate volume changes, shorten electron diffusion pathways, improve the electrode–electrolyte interface, and enhance the structural stability [9,10]. So, it can be envisioned that a hierarchical porous NiCo_2_O_4_ material could combine the merits of not only hierarchical nanostructures, but also porous morphologies.

It is well known that three dimensional (3D) hybrid nanostructures with large surface area and short diffusion path for electrons and ions are promising electrode architectures for high-performance sensor devices. The fabrication of fast, sensitive and selective glucose sensors are in high demand, because glucose detection is very important for patients suffering from diabetes. There are mainly two types of glucose sensors, one is the glucose oxidase-based sensor and other is the non-enzymatic glucose sensor. The glucose oxidase-based sensors are popular due to their high sensitivity and selectivity towards glucose detection and high stability over a wide range of pH, thus different amperometric and potentiometric glucose biosensors have been constructed [11,12]. Amperometric glucose sensors have been developed without the use of enzyme, but potentiometric glucose sensors without the use of enzyme are difficult to construct. The potentiometric technique is simple for measuring glucose level on spot and applicable to quantify the glucose level inside the cell [13]. By exploiting the redox property of binary metal oxides like NiCo_2_O_4_ for the oxidation of glucose molecules, a biosensor is proposed in the present work. Beside this, this study may shed some light on simple and cost effective preparation of hierarchical porous nanostructures and the development of materials with advanced functions for sensor devices.

The crystalline NiCo_2_O_4_ nanostructures synthesized by using hydrothermal method on nickel foam substrate were used as the backbone to support and provide reliable electrical connections to the CoxNi_1−x_ DHs coatings with surface areas accessible to electrolyte, enabling full utilization of the CoxNi_1−x_ DHs and fast electronic and ionic conduction through the electrode. The nickel cobalt oxide nanostructures were characterized by the scanning electron microscopy, X-ray diffraction and X-ray photoelectron spectroscopy techniques. Further these nanostructures were utilized to develop a sensitive, stable and selective glucose sensor using the potentiometric method.

## 2. Experimental Section

Cobalt chloride hexahydrate (CoCl_2_·6H_2_O), Nickel chloride hexahydrate (NiCl_2_·6H_2_O), urea (CH_4_N_2_O), D-glucose, glucose oxidase, ascorbic acid, uric acid, dopamine, sodium hydrogen phosphate, potassium hydrogen phosphate, sodium chloride and potassium chloride were purchased from Sigma-Aldrich (Stockholm, Sweden) and used without any further purification. The nickel foam has been purchased from Goodfellow Cambridge Ltd. (Huntingdon, UK) and used for the growth of NiCo_2_O_4_ nanostructures.

A low temperature aqueous chemical growth method has been employed for the growth of NiCo_2_O_4_ nanostructures on nickel foam as substrate. Initially six pieces (each piece with a length of 3 cm and width of 1 cm) of nickel foam were sonicated in an ultrasonic bath for 20 min in isopropanol, then washed with acetone and deionized water, respectively, and dried by nitrogen gas. A cobalt chloride hexahydrate seed crystal solution was deposited on these pieces by the help of a spin-coater. This procedure was repeated three times at 4,000 r.p.m for 20 s and after that the samples were left for annealing at 100 °C for 20 min. Now for the preparation of precursor solution 2.37 g of cobalt chloride hexahydrate, 1.185 g of nickel chloride hexahydrate and 2.7 g of urea were dissolved in 75 mL of deionized water and then the solution was left on stirring for 30 min. After the completion of the annealing time, the nickel foam pieces decorated with seed particles of cobalt chloride hexahydrate were placed in the beaker containing precursor solution by the help of a Teflon sample holder facing downward and the beaker was kept in a preheated oven at 95 °C for 5–6 h. After the growth period nickel foam pieces with the NiCo_2_O_4_ nanostructures were taken out from the growth solution and washed in the deionized water in order to remove residual solid particles from the surface. Finally, the samples were dried in air at room temperature. Further these nanostructures were annealed at 450 °C for 3 h for the complete conversion of the hydroxide phase into the oxide phase of nickel cobalt.

A 10 mg/mL glucose oxidase solution was prepared in 10 mM solution of phosphate saline buffer solution of pH 7.4. One hundred μL of 2.5% glutaraldehyde was added to the enzyme solution as cross linker in order to prevent the self-enzyme molecular reaction. After obtaining a homogenous solution of enzyme, the nanostructures were dipped in the enzyme solution for 5 min and dried at room temperature. The sensor electrodes were left at 4 °C in refrigerator when not in use.

The morphology and structural properties of NiCo_2_O_4_ nanostructures were studied with a LEO 1550 Gemini field emission scanning electron microscope running at 15 kV. The crystal quality of these nanostructures was studied by X-ray powder diffraction (XRD) using a Phillips PW 1729 powder diffractometer equipped with CuKα radiation (λ = 1.5418 Å) using a generator voltage of 40 kV and a current of 40 mA. The XPS measurements were performed by ESCA200 spectrometer in ultrahigh vacuum with a base pressure of 10^−10^ mbar. The measurement chamber was fixed with a monochromatic Al (Kα) X-ray source using photons with frequency (hv = 1,486.6 eV).

The electrochemical response for the detection of glucose was recorded by Metrohm model 744 pH meter using two electrode systems. Silver-silver chloride was used as reference electrode and the NiCo_2_O_4_ nanostructures as a working electrode. The response time was measured by a Keithley 2400 electrical instrument. All the concentrations of glucose solution were prepared in 10 mM saline phosphate buffer solution of pH 7.4. All the measurements were performed at room temperature.

## 3. Results and Discussion

### 3.1. Characterisation of NiCo_2_O_4_ Nanostructures

**Figure 1.**
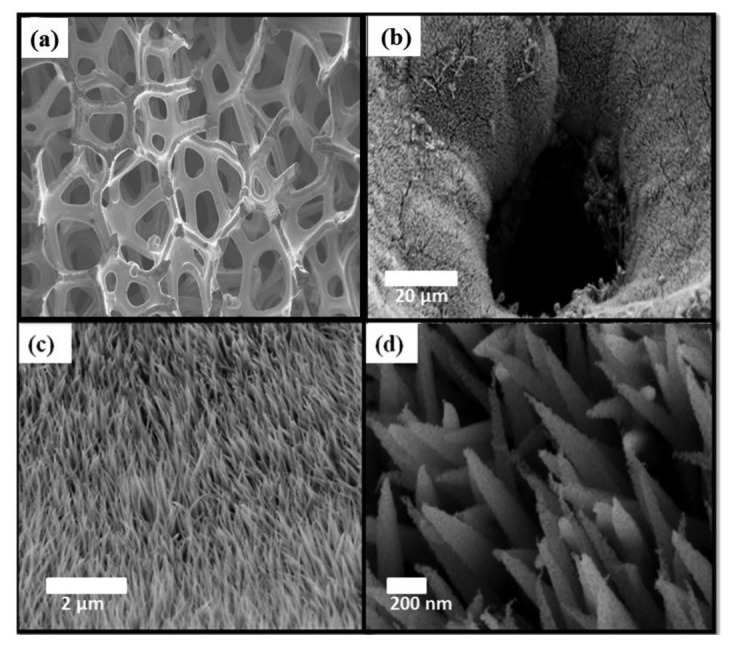
(**a**) SEM image of bare Ni foam substrate (**b–d**) Typical SEM images at different magnifications of NiCo_2_O_4_ nanostructures grown via low temperature hydrothermal method.

The structural study was performed before and after the growth of NiCo_2_O_4_ nanostructures for better analysis of the grown nanomaterial. Figure 1a is the SEM image of bare Ni foam substrate measured at 100 μm.

It is clear from the image that Ni foam is three dimensional and highly porous. The Ni foam has thickness of 1.6 mm with a porosity and purity of 95% and on average there are 20 pores per cm. Figures Figure 1b–d show the SEM images at different magnifications after the growth of NiCo_2_O_4_ nanostructures. Figure 1b shows the SEM image of NiCo_2_O_4_ nanostructures measured at 20 μm and it can be seen that the nanostructures are highly dense and uniform on the nickel foam and composed of nanowires. Figure 1c shows the SEM image measured at 2 μm and it can be seen that top surface of the nanostructures is thin and looks like a needle morphology. Figure 1d shows the high resolution image measured at 200 nm showing a clear view of the needle-like morphology. It also gives a clue that these contain aggregations of nanoparticles, which finally results in the needle-like morphology.

**Figure 2.**
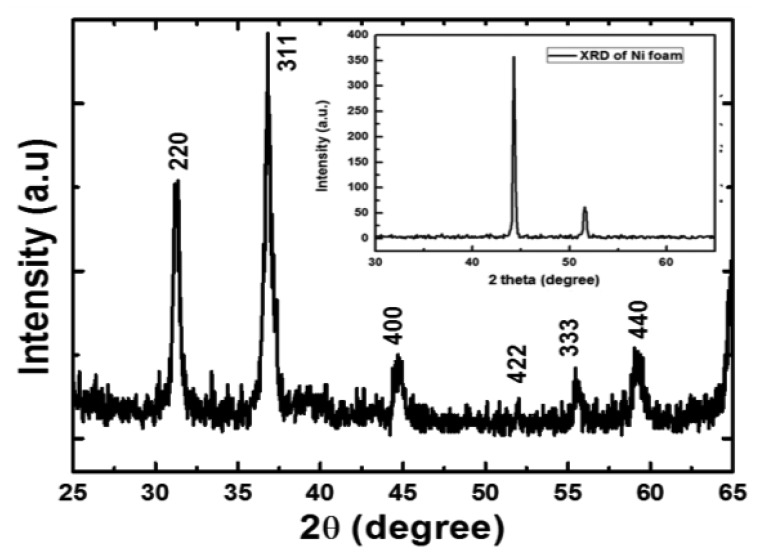
The XRD spectrum of NiCo_2_O_4_ nanostructures and inset is showing XRD spectra of bare Ni foam substrate.

The crystalline study was investigated by XRD before and after the growth of NiCo_2_O_4_ nanostructures. The XRD of bare Ni foam substrate is shown in the inset of Figure 2. The XRD pattern revealed two peaks related to Ni. One is well dominated around ∼44° and the other is relatively low at around ∼51.5°. In Figure 2 the visible diffraction peaks for the respective crystal planes include 220, 311, 400, 422, 333 and 440 (as per JCPDS card no. 73-1702). The entire diffraction pattern peaks could be assigned to the face centered cubic crystalline arrays of NiCo_2_O_4_ with a space group of Fd3m and are according to the reported work [5]. This study indicates the formation of pure phase of NiCo_2_O_4_ nanostructures and no other crystalline phase was observed.

**Figure 3.**
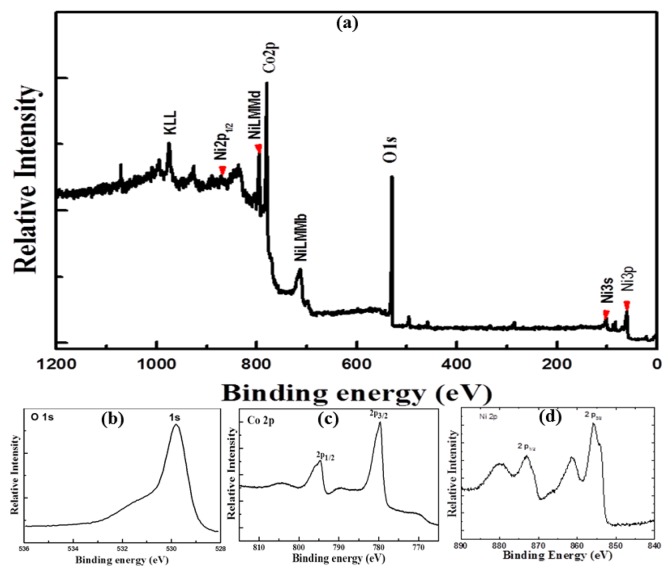
XPS spectrum of NiCo_2_O_4_ nanostructures, (**a**) wide scan spectrum, (**b**) O 1 s spectrum, (**c**) Co 2p spectrum, (**d**) Ni 2p spectrum.

In order to get deeper insight in the elemental composition of NiCo_2_O_4_ nanostructures, X-ray photoelectron spectroscopy (XPS) measurements were performed and the results are shown in Figure 3. In the wide scan spectrum the elements Ni, Co, and O are detected as shown in Figure 3a.

Figure 3b shows the Co 2p photoemission with binding energies of 780.4 eV for Co 2p _3/2_ and 796.2 eV for Co 2p_1/2_, however satellite structure at the binding energies of 786.7 and 802.5 eV for Co 2p _3/2_ and Co 2p_1/2_, respectively, are in good agreement with the reported values [14]. For the O 1s XPS spectrum, the observed binding energy at 529.6 eV is typically assigned to the O-Co/Ni bonding [15] as shown in Figure 3c. The well resolved component energy peak positioned at 531.4 eV is associated to several defect sites with less amount of oxygen coordinated in the sample [16]. Figure 3d shows the Ni 2p _3/2_ peak at 855.1 eV and the measured Ni 2p ½ peak at 873.2 eV is attributed to NiO in the sample [17].

### 3.2. The Potentiometric Response and Working Performance of Glucose Sensor Based on NiCo_2_O_4_ Nanostructures

**Figure 4.**
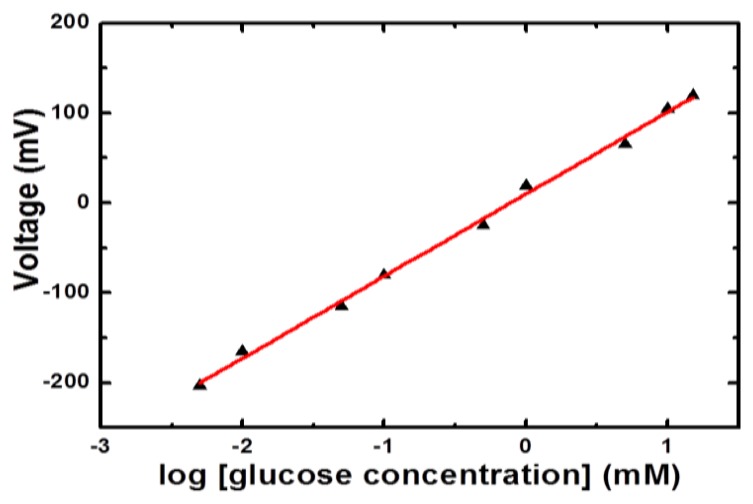
The calibration curve of glucose biosensor based on NiCo_2_O_4_ nanostructures for linear concentration range of 0.005 mM to 15 mM.

Different concentrations of glucose in the range of 0.001 mM to 20 mM were prepared in 10 mM phosphate buffer solution of pH 7.4. The immobilized glucose oxidase-based NiCo_2_O_4_ sensor electrode was inserted in 0.001 mM glucose concentration for measuring the output potential response and it was noticed that sensor showed a response for this concentration. Then the sensor electrode was dipped in 0.005 mM glucose concentration and a steady, stable and strong electrical signal was observed compared to the response for 0.001 mM concentration of glucose. Afterwards the NiCo_2_O_4_- based electrode was used in higher concentrations of glucose and a dominant electrical signal was recorded up to 15 mM of glucose concentration. Beyond 15 mM concentration of glucose a saturation limit was observed so a linear range for the presented glucose sensor based on NiCo_2_O_4_ nanostructures was recorded from 0.005 mM to 15 mM glucose concentrations, as shown in Figure 4.

The detection limit was found to be 1.49 × 10^−3^ mM which is not shown in the calibration curve and the limit of quantification was found to be 1.9 × 10^−3^ mM. The enzyme-based glucose sensors adapt a reaction mechanism catalysed by glucose oxidase that can be represented as follows:

(1) $$H_{2}O+O_{2}+\beta -D-\text{Glucose GOD}\to \delta -\text{Gluconolactone}+H_{2}O_{2}$$

The initial and instant products of the above reaction include δ-gluconolactone and hydrogen peroxide. Either of these two products or the amount of oxygen consumed during the reaction can be useful for the detection of glucose. Due to the presence of water in testing solution of glucose, a spontaneous reaction occurs, which converts the gluconolactone into gluconic acid. Finally at the pH of 7.4 charged species were produced in the solution from the reaction of gluconic acid with water. The positively charged H_3_O^+^ and negatively charged gluconate^−^ were in contact with the immobilized glucose oxidase NiCo_2_O_4_ nanostructures resulting in a mobile surface charge, which helped in the generation of a strong electrical signal from the sensor electrode. The above discussion can be summarized in the following reaction:

(2) $$\delta -\text{Gluconolactone}\to {\text{Gluconate}}^{-}+H^{+}$$

**Figure 5.**
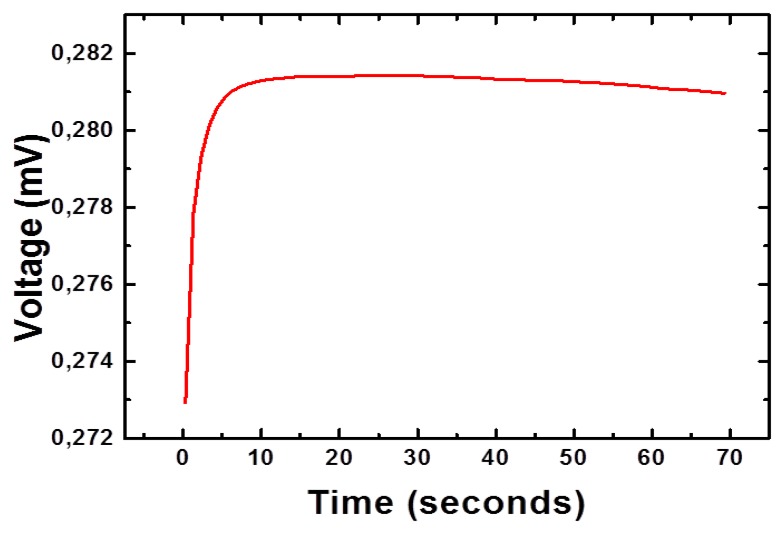
The response time of glucose biosensor in 1 mM glucose concentration.

Moreover, the increase in the amount of H_3_O^+^ ions in the solution lowered its pH, which can also be used for the monitoring of glucose [18]. The change in the concentration of charged species around the working electrode is responsible for the production of the output potential signal [19]. The nickel foam is porous in nature and it remained useful for the growth of three dimensional (3D) NiCo_2_O_4_ nanostructures. The 3D pattern of grown NiCo_2_O_4_ nanostructures further adsorbs high degree of glucose oxidase and resulted in highly sensitive potentiometric glucose sensor with sensitivity of 91.34 mV/decade. In addition to this, the sensor electrode has shown a fast response time of less than 10 s, as shown in Figure 5, which could be attributed to the needle-like morphology of NiCo_2_O_4_ with high surface area on a porous substrate. The high sensitivity and a fast response time for a reasonable range of glucose concentration gave a clear indication for its potential application in the monitoring of glucose concentrations.

**Figure 6.**
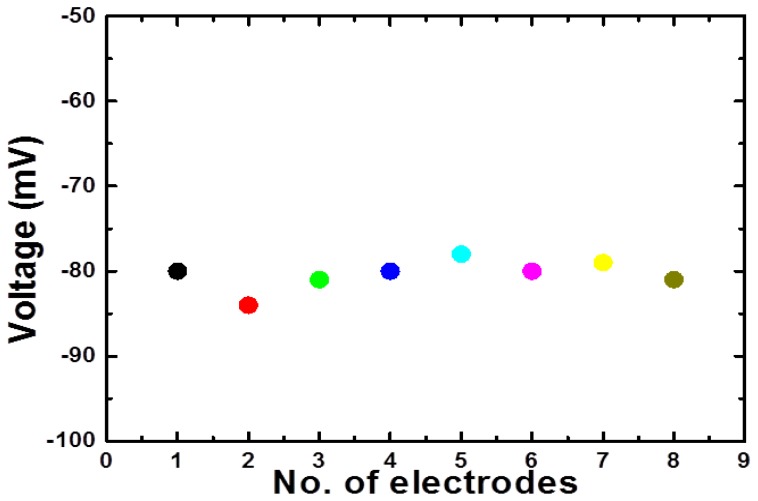
The reproducibility of glucose biosensor in 0.1 mM glucose concentration.

The reproducibility describes the sensor to sensor response for different independent sensor electrodes prepared under similar conditions. Eight independent sensor electrodes were used for the measurement of reproducibility in 0.1 mM concentration of glucose as shown in Figure 6. It can be seen that the sensor to sensor responses deviated less than 3% from each other by relative standard deviation. This demonstrates the highly reproducible response of the proposed sensor based on nickel foam using NiCo_2_O_4_ nanostructures. The stability of the sensor was monitored for three weeks by testing the sensor in the detected range of glucose for every week and it was observed that sensor remained stable by showing the same sensitivity and detection range for that period of observation after being kept at 4 °C in refrigerator when not in use. The common interferents for glucose sensors are ascorbic acid, uric acid, dopamine, *etc.* The sensor electrode was inserted in the physiological concentrations of these interferents present in the human serum and the presented sensor showed negligible response for these interferents due to the fact that glucose oxidase is highly selective for the oxidation of glucose relative to these interferents using potentiometric method. The repeatability of NiCo_2_O_4_ nanostructures based glucose sensor electrode was evaluated by conducting a series of three experiments for three consecutive days.

**Figure 7.**
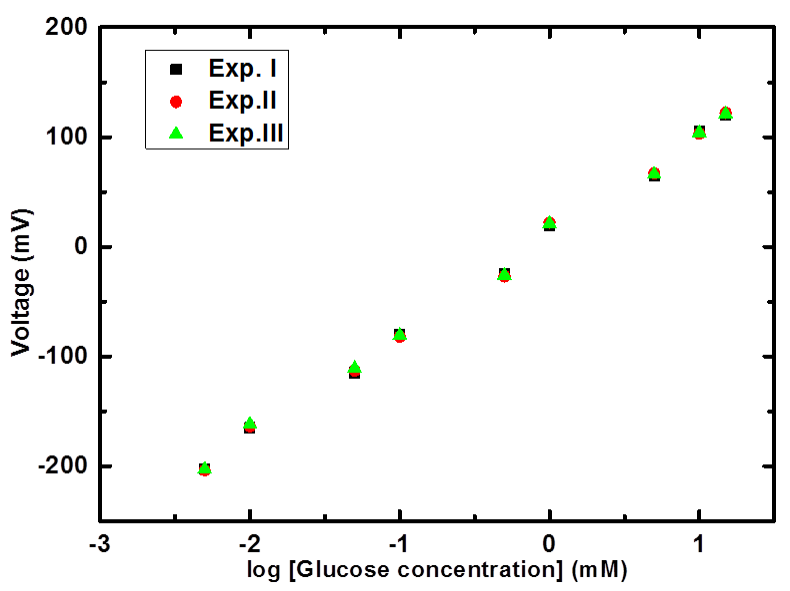
The repeatability curve of the proposed glucose biosensor for 3 consecutive experiments.

The sensor electrode showed excellent repeatable response as shown in Figure 7. This investigation showed that same electrode can be used for a period of three days without any abrupt change in the sensitivity, stability and detection range of glucose concentrations. Table 1 is showing a comparison between the characteristics of the presented potentiometric biosensor and some other previously reported glucose biosensors.

## 4. Conclusions

In this study, 3D nanostructures of NiCo_2_O_4_ were fabricated on nickel foam by the hydrothermal method. The nanostructures were well characterized by the SEM, XRD, and XPS techniques. The nanostructures exhibit needle-like morphology grown in 3D on the porous nickel foam substrate. The XRD data has shown that the material is only composed of NiCo_2_O_4_ nanomaterial. By exploiting the favourable porosity of the prepared nanomaterial, glucose oxidase was immobilized on it for the development of a highly sensitive glucose sensor. The proposed sensor electrode detected a wide range of glucose concentrations from 0.005 mM to 15 mM with a sensitivity of 91.34 mV/decade and showed a fast response time of less than 10 s. Moreover, the sensor electrode showed acceptable reproducibility, repeatability, stability and selectivity. All these obtained results indicate its potential use for the determination of glucose in medical samples, food and other areas.

## Figures and Tables

**Table 1.** Comparison of the characteristics of the presented work and some other previously reported glucose biosensors.

**Matrix**	**Sensitivity**	**Response Time**	**Shelf Life**	**Range**	**Detection Limit**	**Reference**
InN	80 mV/decade	<2 s	14 days	1.0 × 10^−5^ – 1.0 × 10^−2^ M	-	[20]
Multiwall carbon NTs	12.1 μA/mM	-	5 weeks	1–500 μM	1.3 ± 0.1 μM	[21]
Polypyrrole	90 mV/decade	30 min	10 days	6.0 × 10^−5^ – 5.0 × 10^−3^ M	-	[22]
Gold nano particles	2.3 mA/M	<5 s	>2 weeks	1.0 × 10^−6^ – 8.0 × 10^−4^ mol/L	5.0 × 10^−7^ M	[23]
Iodide	65.2 ± 0.2 mV/glucose	1–2 min	∼1 month	1.0 × 10^−1^ – 1.0 × 10^−6^ M	-	[24]
Carbon NTs	602.04 μAmM^−1^cm^−1^	30 s	-	5.0 × 10^−7^ – 1.8 × 10^−3^ M	1.0 × 10^−7^ M	[25]
Silver nano particles	135.904 μAmM^−1^	>10 s	10 days	0.5–50 μM	0.1 μM	[26]
NiCo_2_O_4_	91.34 mV/decade	<10 sec	3 weeks	0.005–15 mM	1.49× 10^−3^ mM	Present work
